# High quality genome annotation and expression visualisation of a mupirocin-producing bacterium

**DOI:** 10.1371/journal.pone.0268072

**Published:** 2022-05-05

**Authors:** Anthony S. Haines, Steve G. Kendrew, Nicola Crowhurst, Elton R. Stephens, Jack Connolly, Joanne Hothersall, Claire E. Miller, Andrew J. Collis, Benjamin D. Huckle, Christopher M. Thomas

**Affiliations:** 1 School of Biosciences, University of Birmingham, Edgbaston, Birmingham, United Kingdom; 2 Manufacturing Science and Technology, GlaxoSmithKline, Worthing, West Sussex, United Kingdom; Newcastle University, UK, UNITED KINGDOM

## Abstract

*Pseudomonas* strain NCIMB10586, in the *P*. *fluorescens* subgroup, produces the polyketide antibiotic mupirocin, and has potential as a host for industrial production of a range of valuable products. To underpin further studies on its genetics and physiology, we have used a combination of standard and atypical approaches to achieve a quality of the genome sequence and annotation, above current standards for automated pathways. Assembly of Illumina reads to a PacBio genome sequence created a retrospectively hybrid assembly, identifying and fixing 415 sequencing errors which would otherwise affect almost 5% of annotated coding regions. Our annotation pipeline combined automation based on related well-annotated genomes and stringent, partially manual, tests for functional features. The strain was close to *P*. *synxantha* and *P*. *libaniensis* and was found to be highly similar to a strain being developed as a weed-pest control agent in Canada. Since mupirocin is a secondary metabolite whose production is switched on late in exponential phase, we carried out RNAseq analysis over an 18 h growth period and have developed a method to normalise RNAseq samples as a group, rather than pair-wise. To review such data we have developed an easily interpreted way to present the expression profiles across a region, or the whole genome at a glance. At the 2-hour granularity of our time-course, the mupirocin cluster increases in expression as an essentially uniform bloc, although the mupirocin resistance gene stands out as being expressed at all the time points.

## Introduction

The majority of antibiotics are produced by Actinomycete bacteria and their genomes have been extensively studied [1]. By contrast, the genomes of Gram-negative antibiotic producers have been much less well-studied and could provide, among other things, important insights into both regulation of expression and metabolic pathways involved in the biosynthetic processes. The ability of what were identified as *Pseudomonas fluorescens* strains to produce antagonistic substances had been observed as far back as the 19^th^ century. However, the first characterisation of the antibiotic produced by one of these strains was published in 1971 by Ernst Chain’s group at Imperial College in London [2], after patents had been filed in the UK and USA by Beechams (which after a series of mergers is now part of GSK). The structures and mode of action of the new compound were elucidated by the group at Imperial College with support from Beechams [3] who also then patented a production method for Lithium Pseudomonic Acid [4]. Since the structure of Pseudomonic Acid suggested that it might be made by joining Monic Acid and 9-Hydroxynonanoic Acid via an ester linkage, the possibility of using mutasynthesis to make new derivatives prompted genetic analysis to map the genes involved. The hope was that mutants defective in producing one of the components would accumulate the other and could incorporate a fed compound related to the missing component. A series of transposon insertion mutants allowed us to map a gene cluster required for mupirocin biosynthesis, but no accumulation of either monic acid or 9-hydroxynonanoic acid was observed [5]. The mutants allowed us to clone and sequence the mupirocin biosynthetic gene cluster, and in collaboration with the groups of Tom Simpson and Chris Willis at the University of Bristol, to propose plausible biosynthetic pathways [6–10]. Studies on the biosynthesis of the related antibiotic thiomarinol by *Pseudoalteromonas* bacteria has also increased understanding of key elements of the biosynthetic process [11, 12]. Beechams had already improved production of mupirocin by traditional strain improvement and had found that the patented mupirocin producer NCIMB10586 was a robust organism for industrial fermentation, so having a good genome sequence is important for further work.

Modern DNA sequencing methods make it relatively straightforward to generate large quantities of data and to assemble whole genomes for bacteria. However, there are issues or systematic artefacts associated with all current sequencing technologies which means that ideally a genome should be assembled by combining two or more techniques–usually one to generate accurate reads over short distances and a second providing long-range information to ensure correct assembly across repeats [13]. In practise many sequencing projects are performed with a single technology, often with a rather limited coverage level—to generate a draft genome covering most of the genome. Annotation is then performed, ideally involving a human expert, but more often using one of a number of automated pipelines alone. The result is that any remaining errors in the sequence (for example, single base indels) are not detected, the start of protein coding regions may not be called correctly and gene naming may propagate names that do not mean anything, or may mean something inappropriate to the context. The vast majority of genomes currently submitted to the databases are not completed to the highest standards, in large part due to the complexities and costs associated with these ‘finishing’ processes. This means that there is a significant need for work towards making these capacities as efficient as possible.

To underpin rational approaches to exploit NCIMB10586, both in mupirocin production and as a potential general host for expression and production of high value products, we have determined the genome sequence and annotated it to a high standard using a pipeline that should be applicable to most other bacterial genomes. Also, since expression of the mupirocin synthesis cluster is coordinated through a quorum-sensing system [14] we generated an RNAseq dataset covering the most relevant period of batch culture. To scan the genome visually for genes with similar expression profiles across a time series we have devised a way to summarise such an RNAseq dataset. The resulting genome profile helps to define co-regulated units and the approach has the potential to be adapted to visualise other expression studies such as responses to particular stimuli.

The genome sequence of strain NCIMB10586 has been deposited in the European Nucleotide Archive as accession PRJEB27737.

RNA-seq data have been deposited in the ArrayExpress database at EMBL-EBI (www.ebi.ac.uk/arrayexpress) under accession number E-MTAB-10255.

The PERL scripts created in this work are available from SourceForge at https://sourceforge.net/projects/misc-genomics-tools/

## Materials and methods

### Bacterial strain

The NCIMB10586 strain analysed was re-acquired from the NCIMB strain collection in order to avoid, as far as possible, mutations that may have occurred over the years during routine laboratory sub-culturing. Because it was previously classified as *P*. *fluorescens* [2], but we now think this is not appropriate (see below), we refer to it as *Pseudomonas* NCIMB10586 throughout.

### Genomic sequencing

*Pseudomonas* NCIMB10586 was grown overnight in LB broth. Genomic DNA was isolated from cells using the Qiagen genomic prep kit (Blood and Tissue) and further purified on Qiagen Tip100 columns. To maximise recovery, genomic DNA was spooled after the alcohol precipitation step using a glass rod. Purity and size were assessed using agarose gel electrophoresis and initially quantified using Nanodrop. Library preparation used SMRTbell Template Prep Kit 1.0. The genome was *de novo* sequenced by GATC Biotech on the PacBio RS platform using DNA Sequencing Kit 4.0 v2 and DNA/Polymerase Binding Kit P6 v2. Data processing using the SMRT analysis suite version 2.3.0 yielded the full sequence as a single contig. Illumina sequencing for error correction was performed by MicrobesNG using Nextera XT protocols and a HiSeq2500 machine. Some additional data for confirmation of apparent errors in areas of low coverage used prior RNAseq reads from Edinburgh Genomics. Illumina reads were aligned to the genome using Bowtie2 [15] and Samtools [16] to create a ‘pileup’ file, which was processed using a Perl script.

### Species analysis

We evaluated the *fluorescens* subgroup phylogeny of *Pseudomonas* species using the concatenated partial 16S rRNA, *gyrB*, *rpoD* and *rpoB* sequence strategy of *Mulet et al*. [17] to determine the position of NCIMB10586 within it. This placed the NCIMB10586 in a well-supported clade with *P*. *synxantha* and *P*. *libaniensis*, with the former showing less divergence (S1 Fig). Using the online Jspecies service JSpeciesWS [18, 19], which uses a full genome alignment strategy, we compared NCIMB10586 against available related genomes.

## Genome annotation

**Fig 1:**
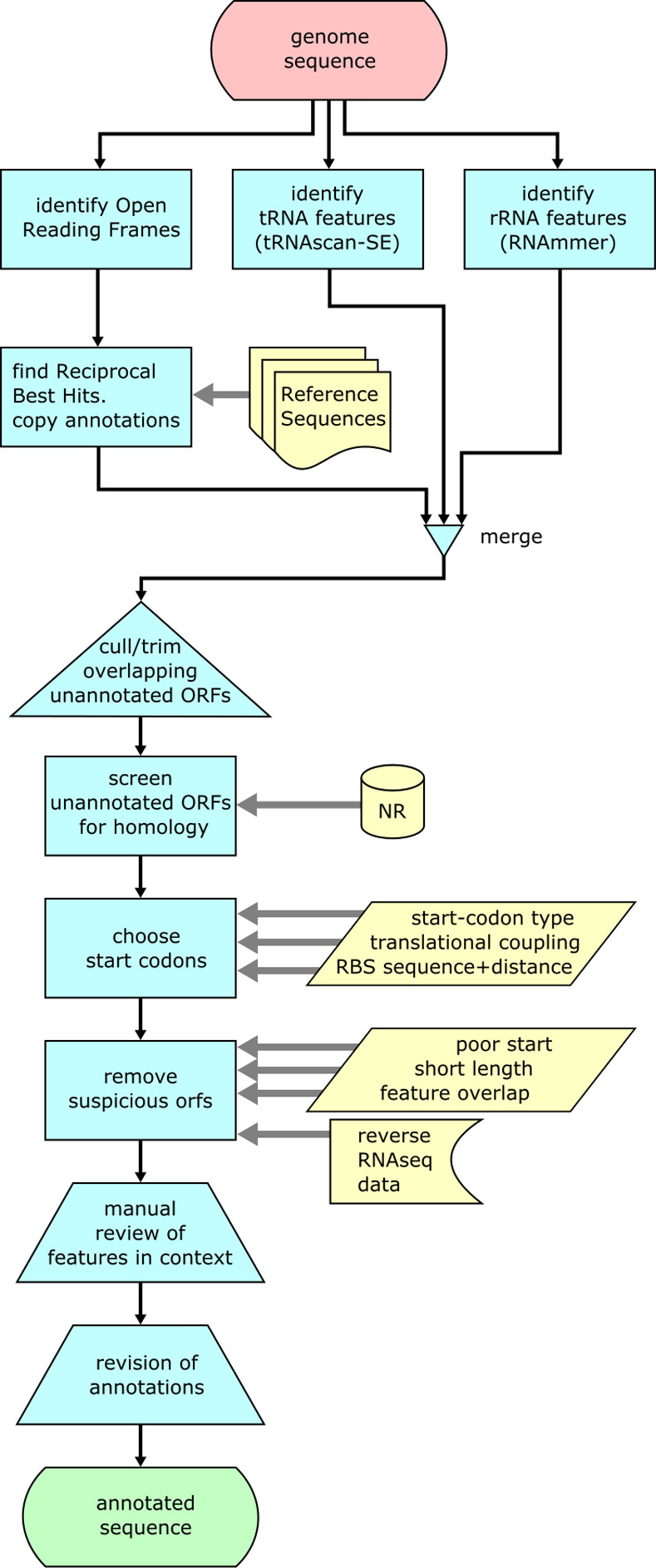
Summary of the pipeline used to achieve a high quality genome annotation.

The annotation pathway utilised, as far as possible, the information inherent in preferred ‘primary’ reference sequences which are both close phylogenetically and of known high quality. Where there was no primary coverage we used lower-quality ‘secondary’ references which were machine-annotated. The whole process of annotation is summarised in Fig 1 and the associated Perl scripts and instructions in their use can be found at https://sourceforge.net/projects/misc-genomics-tools/.

After identifying open reading frames (orfs), reciprocal best hits (RBH) of these were identified against the reference sequences. Non-protein annotations (tRNA and rRNA) were added after identification using tRNAscan-SE [20] and RNAmmer [21]. Orfs overlapping these features were trimmed or discarded. The remaining unannotated orfs were translated and screened against the non-redundant (NR) protein database using BLAST (blastp). Orfs still unannotated were removed. Gaps were identified at the end of this process and any potentially real, completely novel genes recovered.

For each orf, all potential start codons were evaluated to identify the most likely ‘true’ start, with those upstream, or up to 10 codons within a BLAST ‘hit’ used directly. This involved a scoring function that integrates sequence-based clues to the mechanisms involved in initiation of translation, based on data from the literature. To identify potential ribosome binding sites (RBS), the sequence was scanned using the weight matrix based on 2251 sequences from Kibler *et al*. [22] generating a score representing RBS potential for every base position. Each start codon was given an aggregated score over the preceding range weighted on the basis of Chen *et al*. [23] as a symmetric 9-base triangle function with a maximum placing the RBS 7 bases upstream from the start codon (i.e. tvaGGAGnnnnnnnATG). Since ATG triplets are depleted immediately adjacent to start codons [24], the contribution of each RBS sequence was reduced in proportion to the weighted sum of potential start codons ‘visible’ to it. Translational coupling of each potential start codon was evaluated using the model of Tian and Salis [25].

Scores for translational coupling or an RBS were combined with equal weighting and then multiplied by a “start codon type score” dependent on the type (ATG, 16; GTG, 4; TTG, 1) to roughly reflect their relative abundance in bacterial genomes [26, 27]. Each orf was then trimmed to the highest-scoring start codon. Where more than one potential start codon existed, a “confidence ratio” (the highest score divided by the next-highest) was also recorded.

To help remove biologically irrelevant orfs, each orf was assigned a “suspicion” score (the higher the score, the less trusted the orf is to be a real gene) based on start codon strength, orf length, overall RNAseq forward:reverse depth ratio, and overlap with other features. The most suspicious orfs were then removed. Since removal of an orf reduces the score of previously overlapping orfs, this process was iteratively repeated. The remaining orfs were highlighted based on their suspicion level for manual review in Artemis.

### RNAseq

*Pseudomonas* NCIMB10586 was grown in “Starter culture” shake-flasks with medium containing, per litre; 23g yeast extract, 6.5g NaH_2_PO_4_, 5g (NH_4_)_2_SO_4_ and 1g glucose. Flasks were inoculated from different colonies of NCIMB10586, incubated for 15 hours at 25°C and 200rpm, prior to then being used to inoculate final stage 10L fermenters (1% v/v). Fermentation medium consisted of, per litre; 138g glucose, 26g defatted soyflour, 13.9g yeast extract, 11.9g rape seed oil, 4.7g (NH_4_)_2_SO_4_ , 4.6g corn steep liquor, 3.6g NaH_2_PO_4_, 3.3g PPG antifoam and 1.5g MgSO_4_.7H_2_O. Vessels were adjusted to a pre-inoculation pH of 6.8 and then incubated at 22°C, with DO_2_ maintained at 20% *via* agitation and following a natural drop pH control at 5.4 using 70% NH_4_OH.

Three fermentation vessels, set up from different starter cultures, were sampled at two hourly intervals between 6 to 18 hours incubation inclusive. 1ml samples of fermentation broth from 6–12 hour time-points, and 0.5ml samples from time-points thereafter, were mixed with 2 volumes of RNA protect reagent (Qiagen). Cells were collected by centrifugation and frozen at -70°C until processing.

Total RNA was isolated from frozen bacterial samples using the miRNeasy mini kit (Qiagen). RNA was treated subsequently with RNase-Free DNase (New England Biolabs) and re-purified using the RNeasy mini kit (Qiagen). The concentration and quality of RNA in each sample was determined using a 2100 Bioanalyzer (Agilent).

RNA was sequenced at Edinburgh Genomics; 1 lane of HiSeq 4000 was performed, using barcodes to generate 21 libraries with an average yield of 16.8 million paired 75-base reads–the smallest library being 15.4 million paired reads. To produce these, 1 μg total RNA was treated with the Ribo-Zero rRNA Removal Kit (Gram-Negative Bacteria) (Epicentre, now manufactured by Illumina). After treatment the samples were fragmented for 8 minutes. The Illumina protocol was then followed from First Strand Synthesis through to Ligation of Adapters, then enriched using 10 cycles of PCR.

### RNAseq normalisation

The method adopted does not depend on identification of genes but divides both strands of the genome into 100-base ‘tiles’, considered independently. Initially the 75-base, paired fastq read files were aligned against the corrected genome sequence using BowTie2 [15]. The samfiles were processed to determine a read-depth for each tile, collated and written to a csv file, before processing with a further Perl script. A set of preliminary normalisation factors was calculated using these ‘raw’ aligned-read sizes of each library. The least-expressed 50% of the tiles were removed from the process at this point because these should represent the reverse (generally non-expressed) strand of the DNA. Any remaining loci which have a very low depth in any individual library were also discarded to avoid division errors and spurious fluctuations.

As in the IMM derivative of the TMM approach [28], the remaining tiles were then repeatedly analysed to iteratively refine the normalisation factors. In each cycle the list was doubly trimmed to remove those most and least expressed (10% each), and those which vary the most between libraries (80% of the remaining list). Variation was evaluated using the normalised root-mean-square deviation from the geometric mean (NRMSD). For each library, the total unnormalised depth of the remaining tiles was used as the effective library size in order to calculate normalisation factors. This process was repeated until the normalisation factor no longer changed in further iterations.

### RNAseq data visualization

We chose three salient features of a normalised RNAseq dataset representing a series of sampling-points: the total expression, the variation between sampling-points and the peak expression time. Perl scripts were written which form RNAseq read-depths into an easily interpreted display—both as tiles over the entire genome and per-gene—by representing the metrics as channels in the HSL colour model. Lightness uses a logarithmic function of the location’s maximum expression intensity, and ranges from white to mid-grey/colour. Expression levels outside a range (from ‘background’ of double the median reverse tile expression through to the 99^th^ percentile of tile expression) are clamped to the extreme display values. Saturation (colourfulness) is given by a function of the range of expression of the region sampled; S = (max-min)/max expression)^2^. Hue is derived from the inferred peak of expression, linearly adjusted from the peak sample point up to half-way towards a neighbouring sample based on relative expression levels. In our system the hue covers two-thirds of the colour wheel from red through the spectrum to blue—but a range between two primary colours could be used by those with a single-cone colour vision deficiency.

## Results and discussion

### Genome sequencing

It is well recognised that there are advantages and disadvantages for each of the diverse DNA sequencing technologies currently available [29] but we thought it would be useful to quantify the benefits of a hybrid approach. After acquiring PacBio-generated genome sequence of NCIMB10586 as a single 6.3Mb contig, we discovered a single-base deletion in a run of 7 Gs within *mupM*, the product of which confers resistance to the mupirocin cluster [6]. Finding such an error within approximately 75 kb of well-characterised sequence confirmed the need to check for further errors in the remaining sequence, since indels in homopolymer runs are a recognised residual issue in processed PacBio sequences [30]. To determine and correct these, we acquired Illumina reads sufficient to create a draft genome and aligned these against the PacBio sequence using Bowtie2 [15] and Samtools [16] to create a ‘pileup’ file, which was processed using a PERL script to produce a list of non-ambiguous differences. After a manual review these were incorporated into the genome sequence.

Four hundred and fifteen (415) errors were corrected—this corresponds to an error rate in the PacBio sequence of around 1 base per 15kb. Apart from a single base substitution, all of these errors were a single base missing from a homopolymeric run. Our experience is that the presence of such indel errors in base runs now appears to be a common occurrence in PacBio-sequenced genomes in the genome databases. This emphasises the need to be cautious when exploiting such sequences, since a significant proportion of the open reading frames may be disrupted. When our annotation was complete, we found that 266 CDS features (out of 5557; 4.8%) would have been disrupted by one or more errors; 299 errors in total were inside protein coding regions. Although we only annotated 52 of the most obvious transcriptional terminators, three contained a deletion. This likely correlates with rho-independent terminators typically containing very GC-rich sequences, and hence being more likely to carry base runs.

This error correction process required very little additional experimental expenditure for a draft genome’s-worth of Illumina sequencing, but some effort in constructing a pathway to reliably call and correct the errors. While it is true that some errors may have been overlooked in problematic regions due to repetitiveness or low coverage, we consider this step to have significantly increased the overall value of the sequence.

### Species identification and comparison with similar genomes

Inevitably, the annotation of a genome will be influenced by what is already known about similar genomes and so establishing where the organism fits in a phylogenetic tree is useful. In the time since NCIMB10586 was first described [1], species typing within the genus *Pseudomonas* has been considerably refined [31, 32]. Since 16S sequence is recognised to have insufficient discrimination for species-level typing within this genus, we evaluated the *fluorescens* subgroup phylogeny using the concatenated partial 16S rRNA, *gyrB*, *rpoD* and *rpoB* sequence strategy of Mulet *et al*. [17] to determine the position of NCIMB10586 within it. This placed the strain in a well-supported clade with *P*. *synxantha* and *P*. *libaniensis*, with the former showing less divergence (S1 Fig).

Using the online Jspecies service JSpeciesWS [18, 19], which uses a full genome alignment strategy, we compared NCIMB10586 against available related genomes. *P*. *synxantha* BG33R [33], *Pseudomonas* sp. BRG-100 [34] and NCIMB10586 mutually pass the suggested species cut-offs for ANIb, ANIm and TETRA analysis (Table 1). However, *P*. *synxantha* DSM 18928 (which is an alternate designation for the *P*. *synxantha* type strain used in Mulet *et al*. [17], and also for our analysis above) shows considerably less similarity than these cut-offs.

**Table 1:** Relatedness of NCIMB10586 to other genome sequences.

Strain	ANIb ^a^	ANIm ^b^	TETRA ^c^
	% similarity (% match)	% similarity (% match)	
**Primary species-level threshold**	95%	95%	0.999
***Pseudomonas* sp. BRG-100**	98.22 (89.29)	98.78 (89.48)	0.99976
***P*. *synxantha* BG33R**	96.70 (85.46)	97.38 (86.18)	0.99957
***P*. *synxantha* DSM 18928**	89.19 (78.89)	90.67 (77.94)	0.99680
***P*. *libanensis* DSM 17149**	89.30 (78.08)	90.62 (77.60)	0.99664
***P*. *fluorescens* A506**	89.28 (78.41)	90.58 (77.93)	0.99581
***P*. *fluorescens* SBW25**	86.07 (71.61)	88.22 (67.91)	0.98875

^**a**^ ANIb = Average nucleotide identity (Blast)

^**b**^ ANIm = Average nucleotide identity (MUMmer)

^**c**^ TETRA = Tetranucleotide signature frequency correlation coefficient

These discrepancies imply that while strains NCIMB10586, BGR100 and BG33R are closely related, they are probably not *P*. *synxantha* and more work needs to be done to allocate them to a species. Since species of bacteria are in any case a convenience term—ideally guided by relevant phenotypic differences, this question will only be resolved by expert bacterial taxonomists.

### Mupirocin cluster comparisons

Given our primary interest in the genes responsible for synthesis of the antibiotic mupirocin, we compared the mupirocin biosynthetic cluster with those of related strains and this, described in detail in Supplementary Data, will be of great interest to some readers (S1 Appendix in S1 File). *Pseudomonas* sp BRG100, which is being considered for agricultural use as a biocontrol agent of green foxtail, carries an apparently complete mupirocin cluster. Exhaustive comparison of the NCIMB10586 and BRG100 mupirocin clusters revealed no reason why BRG100 should not also make the antibiotic (Table A in S1 File). *Pseudomonas psychrotolerans* strain NS383 carries a less closely related but still collinear mupirocin cluster.

**Fig 2:**
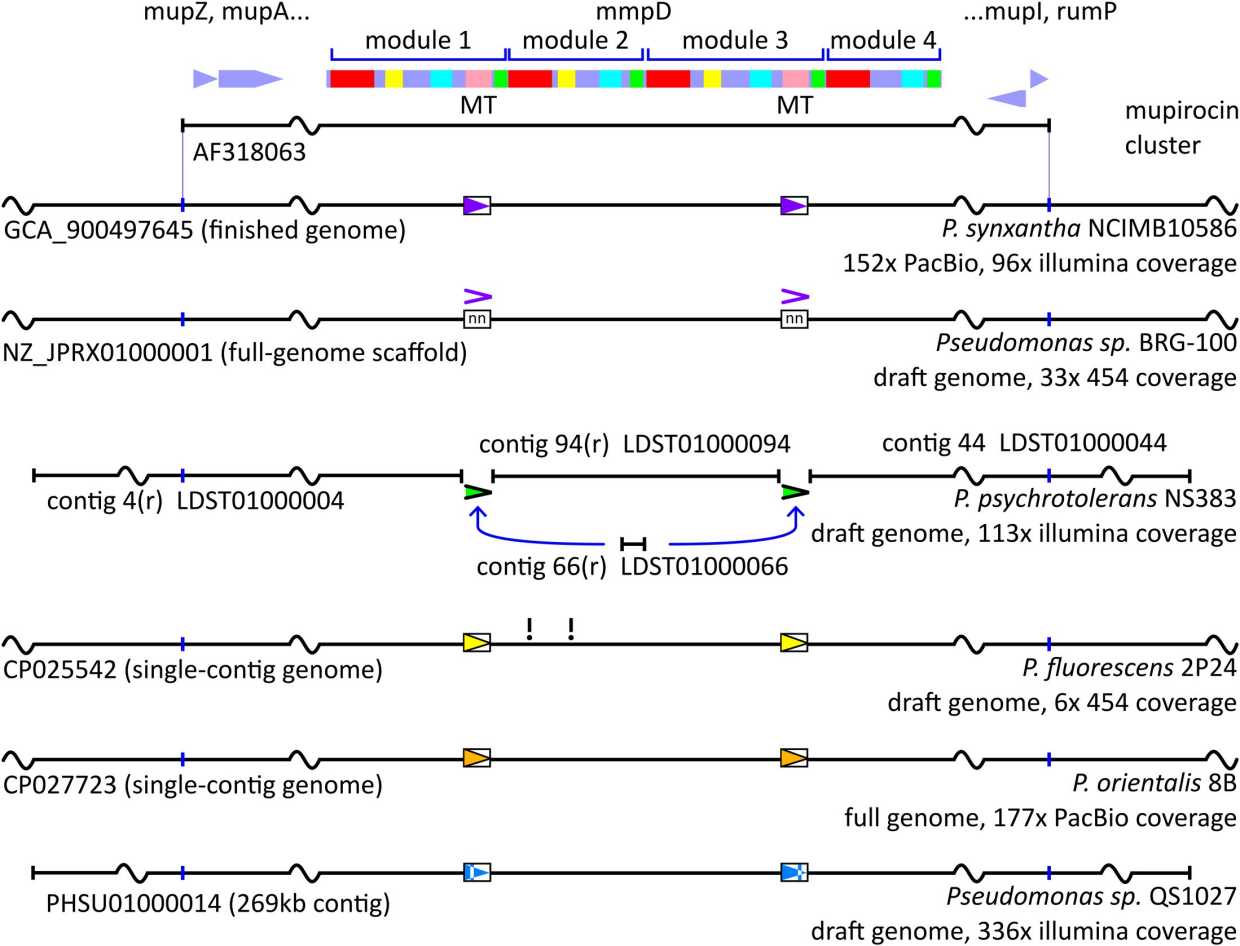
Summary of non-conserved repeats in *mmpD* showing the sequence information available over the mupirocin cluster for several Pseudomonas strains. The drawing is not to scale. A high-identity repeat is present covering methyltransferase domains (indicated in pink at top, labelled ‘MT’) of *mmpD* in NCIMB10586, 2P24 and 8B, and inferred to be present in BRG-100 and NS383, while sequence conservation is lower between the two regions in QS1027. The repeated sequence in each strain is shown as a box with coloured chevron; inferred repeats as a chevron alone. Neither the sequence, nor exact end-point location of these repeats is conserved between strains. Two apparent frameshifts in *mmpD* are indicated (‘!’) in 2P24, however the overall genome coverage is low and these may be artefacts.

Furthermore, all mupirocin clusters found appear to have a repeat sequence comprising approximately the methyltransferase domains of *mmpD* (in modules *mmpD*1 and *mmpD*3), however, the repeated sequence and its exact range (start and end points of the conserved segment) are not conserved between clusters (Fig 2 and S1 Appendix and Table B in S1 File).

### Reference-based genome annotation

We have consistently found that DNA sequences annotated using automated pathways are much less useful than they could be with more human input. We set out to create a hybrid pathway to utilise the information inherent in the most relevant reference sequences, and the other data available, before a final manual pass. The process adopted is summarised in Methods and Fig 1. Favoured (Tier-1) reference sequences used were for the relatively well-studied *Pseudomonas* species strains A506 (accession NC_017911.1), SBW25 (accession AM181176.4) (both related species annotated to a reasonable standard) and the well-studied mupirocin cluster from NCIMB10586 (accession AF318063.3). Tier-2 references were BRG100 (a very closely related machine annotated draft genome, accession JPRX01000001.1) and an automated annotation of the PacBio genome sequence of NCIMB10586 provided by GATC Biotech.

All open reading frames (orf) were ascertained, and then screened against annotations in the reference sequences by determining reciprocal best hits (RBH) [35]. This identifies, for each orf in the genome, whether the best hit in the reference itself has no other, better hit elsewhere in the genome. RBH thus identifies potential orthologues. Stable RNA product features (tRNA and rRNA) were added to the annotation using tRNAscan-SE [20] and RNAmmer [21]. Unannotated orfs overlapping a feature with an RBH or an RNA feature were trimmed to the next start codon or discarded to remove the overlap, very short remnants also being discarded. The unannotated orfs remaining were then translated and screened against the non-redundant (NR) protein database using BLAST (blastp). Orfs still unannotated were removed after this process—but real, completely novel genes would leave gaps which would be identified in a screen at the end of the annotation process, and the feature recovered. In practice we found this to be a rare occurrence.

#### Start codon selection

The orfs remaining after this process carried annotations from at least one of the above sources, tagged with the range and strength of the match. However, in deposited genome sequences we have often suspected that the start of an orf is incorrectly called, so, for each orf we attempted to identify the ‘true’ start codon. (While we are aware that genes can have multiple, alternative start codons, at this point in general they are not annotated, and the prevalence of alternate starts is unclear. We aim to identify the first biologically relevant start codon.) All potential start codons were evaluated, however those more than 10 codons inside the first protein BLAST ‘hit’ were recorded for manual review (where significantly stronger), but not used for automatic trimming. The scoring function used integrates various sequence-based clues to the mechanisms involved in initiation of translation, based on data from the literature.

Ribosome binding sites (RBSs) were identified by scanning the sequence using a weight matrix from 2251 sequences given in Kibler and Hampson [22] with a consensus tvaGGag, noting that the equivalent 3’ 16S rRNA sequence of NCIMB10586 matches that of *E*. *coli*, used in the reference. Scores exceeding the threshold (2.54) are scored at the maximum value, while values approaching this–down to 0.4 lower—are scored linearly down to zero at the bottom of that range. This effectively generates a score representing RBS potential for every base position. In a following stage, each potential start codon is given an aggregated score for RBS over the preceding range, weighted on the basis of Chen *et al*. [23] as a symmetric 9-base triangle function with a maximum placing the RBS 7 bases upstream from the start codon (i.e. tvaGGAGnnnnnnnATG).

It has been observed that ATG triplets are depleted in the region immediately adjacent to start codons [24], presumably due to selection against translation of these alternate reading frames. Given this, the contribution of each potential RBS sequence was reduced to the proportion of the putative start’s contribution to the weighted sum (products of codon type as described below & location weighting) of potential start codons ‘visible’ to it. We found that this down-weighting of RBS with potential off-target initiations changed the start codon identified for 24 of 5557 CDS features in the final annotation.

We also evaluated translational coupling of each potential start codon using the model described by Tian and Salis [25] with a maximum predicted where the start codon is at position -4 in relation to the stop codon (i.e. the last two bases of start codon overlap the stop codon). A linear drop-off is predicted where the start codon is otherwise upstream of the stop codon, from 8/22 of maximum at position -7 to zero at position -25, and for positions -1 and downstream an extended drop-off, from 8/22 of maximum, with a halving distance of 100 bases.

Since genes with a match to just one of the two above structures (translational coupling or an RBS) are common, the score from these are simply combined with equal weighting. This value is multiplied by a “start codon type score”. The strength of a start codon is dependent on the type (ATG, GTG or TTG). In *E*. *coli*, these are present at rates of 83%, 14% and 3% respectively [26]. The rates at which these are used is reported to vary across the bacterial kingdom (and by identification method) with an average of ATG 80.1%, GTG 11.6%, TTG 7.8% [27]. We do not believe it to be desirable to overfit these ratios, and still want to detect weak start codons. Therefore, we used expedient values of 16, 4 and 1 respectively (equivalent to ATG 76.2%, GTG 19.0%, TTG 4.8%) for these scaling factors, which gave acceptable results in practice.

Each orf was then trimmed to the highest-scoring start codon. For evaluation purposes, where more than one potential start codon existed, a “confidence ratio” was also recorded, being the highest score divided by the next-highest.

#### Selection between putative genes

The above procedure was sufficient to reliably identify protein-coding regions, and avoid including non-coding sequences, in large tracts of the sequence. However, in areas where no close homology to the reference sequences exists, multiple orfs may be annotated. In the sequence databases there is enough independently annotated data that there are sometimes competing annotations in different reading-frames. In some cases all can look plausible without independent confirmation. In an attempt to deal with this, we assigned each orf a “suspicion” score (the higher the score, the less assurance there is that the orf is a real gene). We assigned zero, one or two points for each of: start codon strength, orf length, overall RNAseq forward:reverse depth ratio, and overlap with other features. The point cut-offs were determined empirically. After removing the most suspicious orfs, we iteratively repeated this process (removing an orf reduces the score of previously overlapping orfs), progressively reducing the maximum permitted score down to 5. The remaining orfs were high-lighted based on their suspicion level for manual review in Artemis.

The entire genome was then screened manually for the remaining issues. This utilised early versions of the multi-condition RNAseq visualisation described in the section below. Due to an error or omission in processing, a number of very short trimmed termini of much larger orfs were found (and easily removed). The majority of the time was spent on a few edge cases with conflicting evidence. In a few awkward cases the CDS start-codon was revised (it is likely that in some of these cases both start codons are utilised), or a gene was added after reviewing all the data available for the region.

### RNAseq normalisation

Since RNAseq analysis is increasingly common, a major aim of our work was to develop a way to present the results of RNAseq expression profile analysis so that one could easily scan the genome for transcriptional units with similar expression profiles. However, this requires comparison of gene expression by RNAseq across a number of conditions or time points which in turn requires a robust normalisation process. This effectively needs to generate a scaling factor for each library such that the reported expression level of a hypothetical perfectly constitutive and therefore identically expressed sequence would be the same for all libraries. Simply scaling libraries based on aligned read count does not achieve this since, for example, an increase in a subset of genes alone would down-scale genes which retained the same level of expression.

A variety of different ways of normalising RNAseq data exist [36]. For our purposes, these had a number of drawbacks: many only compare conditions in a pairwise manner (e.g. TMM), and also typically have an ‘atomic’, gene-based model (where each read-pair is treated as a single indivisible unit and assigned to a gene in its entirety—or discarded entirely, as in DESeq). Pairwise comparison is problematic if different subsets of genes are differentially regulated in 3 or more conditions under test–which is the expected case for all sets of conditions varying on more than one simple axis. A gene-based analysis has at least two issues: errors in annotation affect the result, and in prokaryotes the unit of consideration is more properly the operon; assigning reads as indivisible entities to discrete genes can introduce artefacts.

The strategy we used is based on the assumption (similar to TMM and DESeq methods) that a significant proportion of genes are expressed at a largely constant level and are relatively evenly expressed in all samples.

We designed an approach which does not depend on identification of genes, by dividing both strands of the genome into 100-base ‘tiles’, considered independently. Initially the 75-base, paired fastq read files were aligned against the corrected genome sequence using BowTie2, and the read-depth calculated for each tile. A set of preliminary normalisation factors was calculated using these ‘raw’ aligned-read sizes of each library. The least-expressed 50% of the tiles were removed from the process at this point because these should represent the reverse (generally non-expressed) strand of the DNA. Any remaining loci which have a very low depth in any individual library were also discarded to avoid division errors and spurious fluctuations.

**Fig 3:**
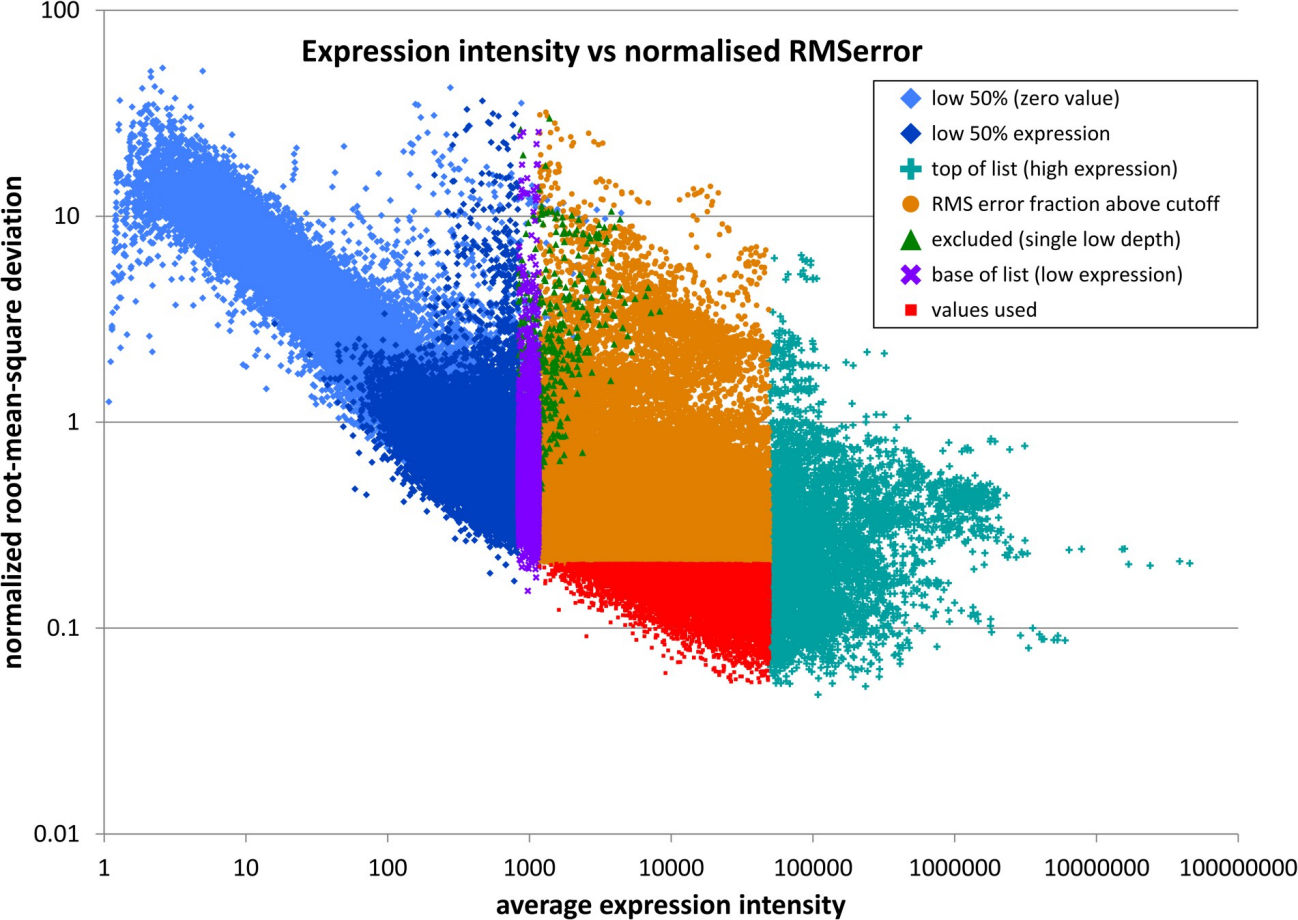
Plot of tile expression intensity vs a measure of variation ([root-mean-square deviation]/mean expression) categorised as determined using the final normalisation factors. For display in this graph only, expression intensities of zero have been substituted by one to permit calculation of the geometric mean.

As in the IMM derivative of the TMM approach [28], the remaining tiles were then repeatedly analysed to iteratively refine the normalisation factors. In each cycle the list was doubly trimmed to remove those most and least expressed (10% each), and those which vary the most between libraries (80% of the remaining list). Variation was evaluated using the normalised root-mean-square deviation from the geometric mean (NRMSD). For each library, the total unnormalised depth of the remaining tiles was used as the effective library size in order to calculate normalisation factors (Fig 3). This process was repeated until the normalisation factor no longer changed in further iterations.

In the TMM method, the selected samples are weighted based on their variability in expression. The above procedure does not do this, since the NRMSD metric we use to determine variation over more than two libraries is not suitable for this. Nevertheless, this procedure did produce an effective set of scaling factors.

### Visualisation of multi-condition RNAseq genomic data

A major aim of our work was to identify transcriptional units that had similar expression patterns over time and particularly those that were similar to the *mup* cluster. This prompted us to consider how visualisation of the expression profile across a time series could be incorporated into the genome map. We had 7 sample points in each of three replicate time-series and needed to process this data into a form in which the important differences are clearly presented.

One prerequisite for interactively viewing the data is sequence browser software. Artemis [37] is a widely used, freely accessible tool which shows the six-frame translation and various analyses in context. However, while Artemis and other viewers can display RNAseq read data directly, we have found this to be computationally cumbersome due to memory issues. Artemis can also display custom data as a heatmap—for example, we formatted our data into an approximately 400Mb file representing the read-depth per base of each time-point, on both strands. However, while this is useful, we felt that an even more condensed representation might give more rapid insight into the structure of the data.

The approach used here did not require modification of the viewing program itself. Instead, our strategy incorporated this data by encoding it as misc_feature annotations with colour qualifiers, which are rendered in one of two strips along the sequence, dependent on orientation. Using Artemis in this manner, the display is essentially restricted to a colour bar in each direction for each region. This method leverages the utility of Artemis, and permitted experimentation and rapid iteration during development. Although our approach here is ideally suited to samples from a time-series or other ‘involved’ axis rather than a diverse set of conditions, we believe that the same principles should be adaptable to displaying other types of datasets.

RGB (Red Green Blue) is the additive colour model usually used internally to describe colours to be displayed by modern computer screens. This has 3 channels representing the detection peaks of the three colour receptors of human vision. While two-channel data is reasonably straightforward to interpret, a visualisation using all three RGB channels directly to represent different properties is not easily parsable. As an alternative we chose HSL (Hue, Saturation–approximately “colourfulness”, and Lightness) which is a relatively simple colour model that attempts to represent colours in a way they are easily thought of. One potential disadvantage of using this model is that visual clarity of the three variables is not independent: if saturation is low hue cannot be discerned, and if lightness is high or low both hue and saturation may be unclear. However, although it may be that in theory more information could be presented in a different encoding scheme, we feel that HSL does allow scope to identify and extract the three most useful parameters to represent the RNAseq profile at each locus.

Initial analysis of the profiles of the time series from our three replicate fermentation vessels showed that they were remarkably similar. This suggests that culture conditions were standardised well enough that they grew congruently, so there was little value in trying to visualise the variation between them. We therefore merged the three (normalised) libraries at each time-point. This left a 7-datapoint profile to be represented in three parameters. It is obvious that these cannot completely fit an arbitrary profile, but fortunately, most read-depth profiles are essentially points on a smooth curve.

Expression intensity is the obvious primary variable in RNAseq data. The logarithm (plus 1) was used because intensity has a large dynamic range. Mapping this to lightness (L) makes sense, since whatever other parameters are reported, they will not matter if expression is low. Using the maximum expression of any time-point gave a slightly clearer display than an average of all time-points. Across the entire genome, we observed the presence of a low level of ‘noise’ reads, which may represent DNA contamination or ‘random’ transcription initiation events. Combined with the log scale this means that even sequence that does not appear to encode a transcriptional unit may show some signal. To achieve a clearer display we subtract this background signal from each feature’s intensity. To determine a reasonable value for this noise removal, we reasoned that the vast majority of the genome is transcribed on one strand only. We therefore calculated the per-tile average intensity of the least-expressed strand for every locus and identified the median value. We found that double the median is a suitable value for noise suppression. Because the distribution of gene expression strengths is positively skewed (with many weakly expressed genes and a long tail of relatively few, strongly expressed genes), we use the 99^th^ percentile intensity of the most-expressed time-point as the upper limit. In our data this shortens the logarithmic scale by almost a quarter, improving discrimination of the majority of the data.

**Fig 4:**
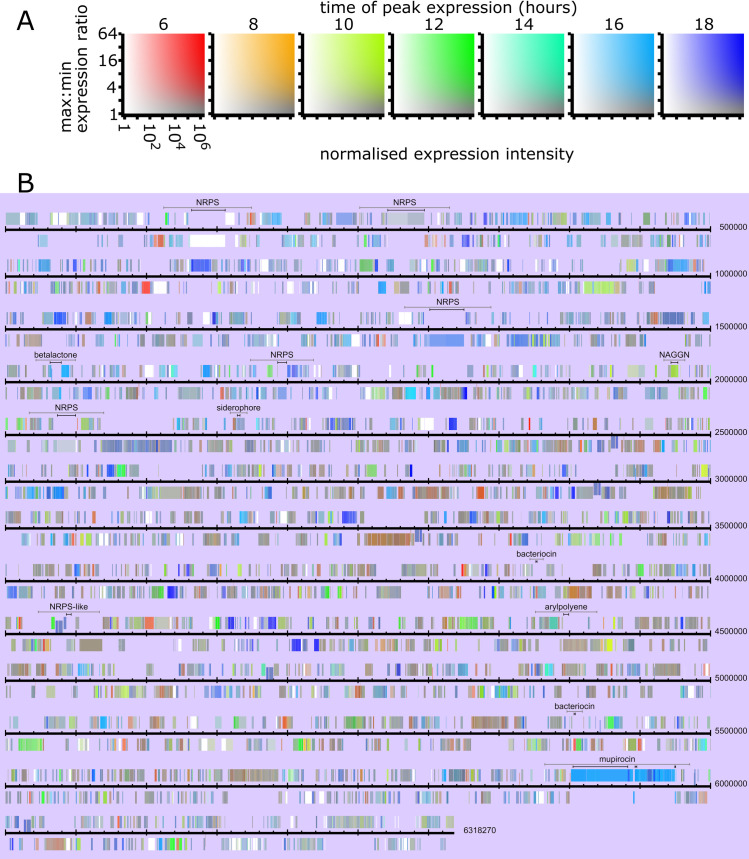
Visualisation of expression over the NCIMB10586 genome. A) Key. The colour of a feature depends on time-point of highest expression (hue), expression intensity (lightness; x axis) and ratio of maximum to minimum expression (saturation; y axis). So: white = consistent low expression; grey = high expression, little variation; intense colour = high expression and big variation between highest and lowest level. B) Features are shown above or below the DNA line dependent on the coding strand. Protein-coding regions are shown as coloured boxes slightly separated from the DNA line. RNA features are shown as coloured boxes adjacent to the DNA line. Putative clusters identified by antiSMASH are shown: grey bar–neighbourhood; black bar(s)—protocluster regions.

The HSL colour space can be considered as a graduated cylinder with black and white at the ends, grey along the central axis, and pure colours around the central circumference. While initially we considered light intensity as a direct analogue for read depth (i.e. black as zero expression), we later realised that this is sub-optimal for print, since the colour inks are masked by black at higher coverages. Therefore, we now use just the ‘light half’ of the colour space, with white as zero expression and mid-grey to full saturation colour as high expression (Fig 4A).

The second parameter we selected was time of maximum expression, which we mapped to hue (H), covering two-thirds of the colour circle (the spectrum from red to blue) to avoid confusion over values at the extremes. Although we had only 7 time-points, we found the apparent resolution could be improved by adjusting the hue up to halfway towards the adjacent time-point(s) as a linear proportion of their relative intensities to the peak (Fig 4A). Although we recognise that this output is effectively unusable by anyone who is colour-blind, the utility gain is otherwise significant. We believe that using a linear scale of hue between two colours would give an acceptable fall-back solution for those who are not fully colour-blind.

For the remaining parameter we show the square of the range of expression as a proportion of the total; S = (max-min)/max expression)^2^. This has the advantage of simplicity, and also usefully suppresses the display of hue (time of max expression) where it is generally considered not to be significant (changes of less than 2-fold). Although we investigated a logarithmic scale, effectively giving each 2-fold increase in range equal weighting, the above function accentuates differences over the most relevant range of variation (2 to ~20-fold) and hence we think it is superior.

There is a trade-off between resolution along the sequence and storage/computational requirements in the sequence viewer. For viewing the 6.3 megabase NCIMB10586 genome in Artemis, we found 100-base tiles to be acceptable for some purposes, although shorter tiles are preferable where resolution is important. When including an annotation of the read depth profile, 100-base tiles added about 22 Mb to the 16 Mb annotated genome flatfile. A 50Mb file with 50-base tiles could easily be displayed on a desktop PC alongside the 400Mb depth heatmap. For comparison the same PC struggled to cope with 10% of the reads represented as a 1.8 Gb bam file in the IGV viewer.

One potential issue with this display is that the colour model is not perceptually uniform–for instance blue is a much darker colour than yellow, yet HSL treats them as equivalent. This means that the expression level of two areas with different time-point maxima cannot reliably be visually compared. This could potentially be resolved using one of the more complicated, perceptually accurate colour models.

While perusing the genome, we observed several bi-colour operons–with the hue ‘randomly’ switching between two quite distinct tints over a long region. Examination of these uniformly revealed a profile with two peaks of similar strength. While this is not surprising in the sense that only unimodal profiles are well-described by the three parameters selected, we had not expected many operons to show such tightly balanced peaks in expression. Particularly if the resolution is high, it may be possible to reveal more of the hidden variation using error diffusion dithering to ‘stipple’ the output.

When applied to visualisation of a whole genome the profile colour can be incorporated into the depiction of the orfs themselves as demonstrated in Fig 4B which shows the complete NCIMB10586 genome. In Fig 4B the *mup* cluster (5,900,000 to 5,976,000) stands out as a distinct block because of its coordinate regulation. Within this block, the most biologically significant variation is of *mupM* and *mupN*, which are the only genes expressed early in the culture to any great extent. This nuance is not obvious in the plot (especially not at genome scale) as expression also increases co-ordinately with the rest of the cluster.

**Fig 5:**
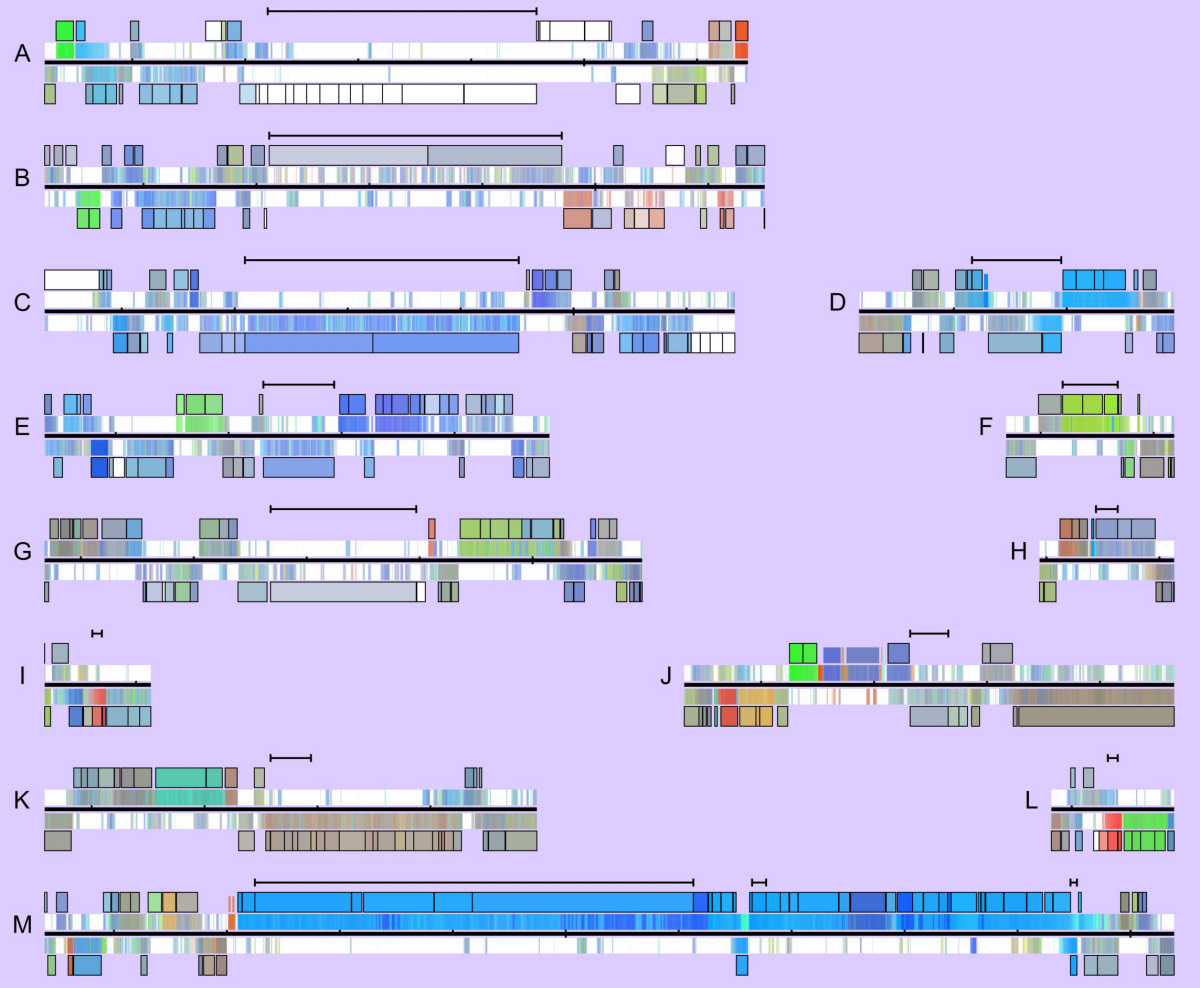
Enlarged view of expression over each cluster in the NCIMB10586 genome identified by antiSMASH. A, B, C, E, G - NRPS; D - Betalactone; F - NAGGN; H - siderophore; I, L - bacteriocin; J – NRPS-like; K - Arylpolyene; M – mupirocin cluster.

The colour profile allows the genome to be very quickly visually scanned for other loci with similar expression characteristics. AntiSMASH v5.2 [38] predicted the presence of twelve other secondary metabolite biosynthesis gene clusters (indicated in Fig 4B and shown enlarged in Fig 5): Five NRPS and one NRPS-like fragment, a beta-lactone containing protease inhibitor, an N-acetylglutaminylglutamine amide cluster, a siderophore, two bacteriocin/RiPP clusters, and an aryl polyene cluster. Of these, most are effectively ‘off’, or have an almost constant low level of expression, in line with the fact that many secondary metabolite gene clusters are silent under standard lab growth conditions [39]. Both putative bacteriocins have moderate expression at the earliest time-point (6 hours) and then lower level as density rises, suggesting possible repression in response to quorum signals [40] which is distinctly different from the other clusters in Fig 5. Using the visualisation (Fig 5) it was in most cases easy to see the likely extent of the cluster, with putatively relevant genes clearly having a distinct expression profile.

The sequence and expression profile is rendered as in Fig 4, with the following alterations: 1) the scale is five times larger–the region shown for the mupirocin cluster (M) is 100kb. 2) Close to the DNA line, the expression profile of the entire range is shown in both directions, drawn from 50-base ‘tiles’. 3) The range and average profile of each protein-coding region is now shown with a black border. RNA products are shown in-line with these but without a border. Black bar(s) above the plot show protocluster regions identified by antiSMASH.

## Conclusions

This paper describes a combination of approaches to achieve a high-quality annotated genome sequence for an important organism. We hope that the eventual quality of the NCIMB10586 genome sequence and the bioinformatics methods used can be of value in improving the sequence quality and annotation of other genomes.

The discovery of increasing numbers of organisms carrying the mupirocin biosynthetic cluster is intriguing especially since the cluster is found in various genomic contexts despite apparently not being associated with features defining a mobile genetic segment that could facilitate Horizontal Gene Transfer. All mupirocin clusters we have seen are collinear, despite significant sequence-level divergence. We have also identified a pair of regions encoding methyltransferases which are routinely highly similar to each other within each mupirocin cluster—despite an absence of increased sequence conservation over this range between divergent clusters, implying the existence of a process which has yet to be explained.

Finally, we have developed a strategy to present an expression profile across a growth time course or series of conditions in an easily comprehended way that can underpin both real-time review in a sequence browser and global characterisation of a genome. We believe that as it becomes easier to generate larger expression datasets, this is only going to become more useful. Since the underlying data of our profile is essentially as compact as a heat-map with just three rows per strand, and could be developed or modified and used for various purposes, the ability to display a colour user-data plot would seem to be a desirable feature in a sequence browser.

## Supporting information

S1 Fig Phylogenetic tree of concatenated partial 16S rRNA, *gyrB*, *rpoD* and rpoB sequences. (PDF)

S1 File Mupirocin cluster comparison with other similar sequences. (PDF)
