# Supplementary material for: High quality genome annotation and expression visualisation of a mupirocin-producing bacterium
Source: PLoS One. 2022 May 5;17(5):e0268072. doi: 10.1371/journal.pone.0268072 (PMC9070926; doi:10.1371/journal.pone.0268072)
Supplement: S1 Fig — (PDF) [file pone.0268072.s001.pdf]

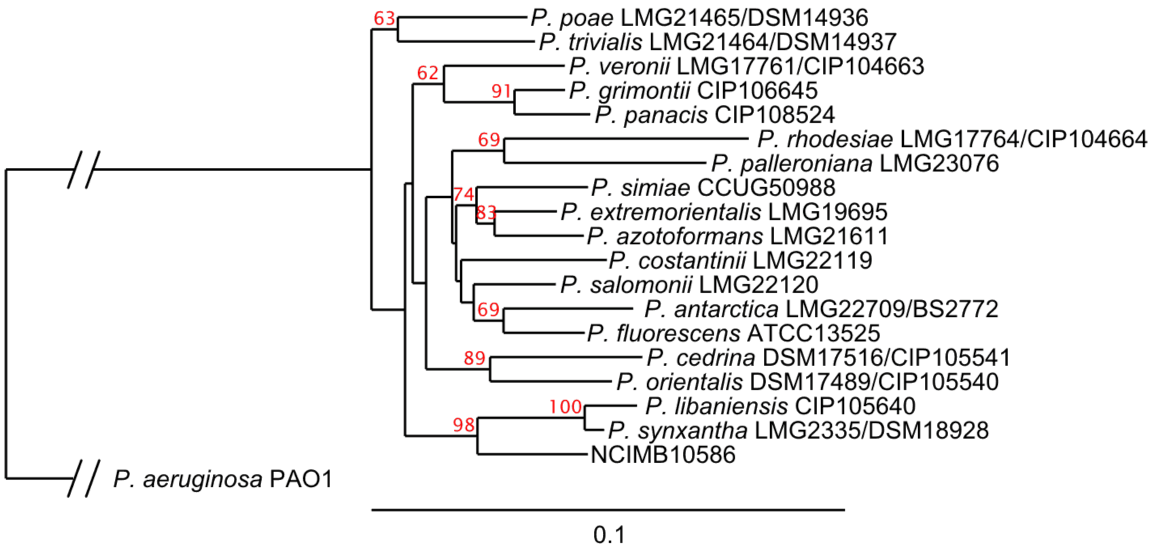

S1 Fig. Phylogenetic tree of concatenated partial 16S rRNA, *gyrB*, *rpoD* and *rpoB* sequences. Sequences were acquired from the nucleotide databases, using BacDive (Söhngen *et al.*, 2016)[1] to identify synonymous database IDs where necessary, trimmed to length, and concatenated as described in Mulet et al., (2010)[2]. The full phylogenetic tree pathway (MUSCLE , Gblocks, PhyML and TreeDyn) was performed using phylogeny.fr (Dereeper *et al.*, 2008)[3]. Numbers in red are the percentage of 100 bootstrap replicates which support the clade.

1. Söhngen C, Podstawka A, Bunk B, Gleim D, Vetcininova A, Reimer LC, Overmann J. BacDive - the bacterial diversity metadatabase. Nucleic Acids Research 2016; 44 (Database issue): D581?D585; doi:10.1093/nar/gkv983

2. Mulet M, Lalucat J, García-Valdés E. DNA sequence-based analysis of the *Pseudomonas* species. Environ Microbiol 2010; 12:1513–1530.

3. Dereeper A, Guignon V, Blanc G, Audic S, Buffet S, Chevenet F, Dufayard JF, Guindon S, Lefort V, Lescot M, Claverie JM, Gascuel O. Phylogeny.fr: robust phylogenetic analysis for the non-specialist. Nucleic Acids Research 2008 Jul 1;36(Web Server issue): W465-9.
