# Supplementary material for: High quality genome annotation and expression visualisation of a mupirocin-producing bacterium
Source: PLoS One. 2022 May 5;17(5):e0268072. doi: 10.1371/journal.pone.0268072 (PMC9070926; doi:10.1371/journal.pone.0268072)
Supplement: S1 File — (PDF) [file pone.0268072.s002.pdf]

S1 Appendix.

Mupirocin cluster comparison with other similar sequences

Over the last few years, a number of sequences homologous to the NCIMB10586 mupirocin cluster have been present in genomes uploaded to the nucleotide sequence databases, in various states of completion. Here we exhaustively compare the most similar of these and compile and investigate certain properties of the others. In particular we find they are all collinear at the gene level. The sequences involved and the sequencing chemistry and read depths are summarised in Figure 2, main paper.

Highly similar Mupirocin cluster in BRG-100 draft genome

Pseudomonas sp. BRG100 (NZ_JPRX01000001) carries an apparently complete mupirocin cluster despite being considered for agricultural use as a biocontrol agent of green foxtail. To assess whether this is likely to encode a functional pathway and synthesise mupirocin we therefore exhaustively compared the differences between the NCIMB10586 mupirocin synthetic cluster and the equivalent sequence of BRG100. Excluding what we infer are repeated sequences (see below) masked out of the published data, there are 231 SNPs, 2 two-base polymorphisms (covering 3 codons), and 2 small indels (Table A in S1 Appendix.). As expected, the large majority are synonymous, and unlikely to affect mupirocin production. Of the 27 'radical' changes that alter the encoded amino acid, 15 were inside a domain (as opposed to in a "linker" peptide), but alignments with similar sequences in Genbank indicated they are within the normal range of variation. Similarly, the intergenic SNPs were examined for overlap with relevant motifs. Overall, this screen found no evidence that the mupirocin synthesis cluster of BRG100 would be non-functional.

Table A. Base-change differences between the mupirocin clusters of NCIMB10586 and BRG100.

Substitution type	SNPs and 2-base polymorphisms
**Synonymous**	159
**Conservative** [a]	32
**Semi-conservative** [b]	9
**Radical**	27
**Intergenic SNP**	7

a Non-negative values in BLOSUM90

b. Non-negative values in BLOSUM65 (where not included in BLOSUM90)

Mupirocin cluster in 2P24 draft genome

The genome of *P. fluorescens* 2P24 (CP025542.1), a rhizosphere bacterium that produces 2,4-diacetylphloroglucinol and suppresses soilborne plant diseases [1], also has a mupirocin cluster. Although this has a number of apparently dysfunctional mutations – such as two frameshifts in *mmpD*, the reported read-depth of this genome is low and we suspect them to be sequencing errors.

Pseudomonas psychrotolerans strain NS383

During a routine Blast search, homologues of several mupirocin cluster genes were also identified in the draft genome of *Pseudomonas psychrotolerans* strain NS383. Further investigation identified four contigs (accessions LDST01000004, LDST01000044, LDST01000066, LDST01000094), apparently comprising a complete, colinear mupirocin cluster. There does not seem to have been any expectation that this strain could produce mupirocin, or recognition of the cluster in the original study [2].

Alignment against the mupirocin cluster sequence suggests that there are no repeated or missing bases at the junctions; the sequence is in four pieces apparently due to a repeated sequence in *mmpD*, both copies of which have been collapsed into a single contig (see Figure 2, main paper). Although we therefore do not know the sequence of either repeat with certainty, we can infer that they must be similar enough (over the length of a typical amplicon in the library) to frustrate the assembly of these repeats and the adjoining sequences into one contig. We have therefore reconstructed the full putative cluster sequence for the purposes of comparison.

The gene order of the *P. psychrotolerans* mupirocin cluster is identical to that of NCIMB10586. However, the nucleotide sequence similarity is surprisingly low – only 68.5% identity (3.1% gap), with an overall concatenated protein identity of 66.1%. Comparison of the two sequences proved useful in studying the regulatory elements of the cluster, which will largely be reported elsewhere.

Mupirocin clusters all bear methyltransferase repeats despite divergent sequence

Curiously, there is also a high-identity repeat in the NCIMB10586 mupirocin cluster over approximately the same regions as those splitting the *P. psychrotolerans* NS383 mupirocin cluster (comprising approximately the regions encoding the methyltransferase (MeT) domains of modules 1 and 3 of MmpD (Figure 2, main paper).) After aligning the repeats of these two *mmpD* homologues, and trimming the range to a region in common, it is clear that the sequence itself is not conserved between clusters (in NCIMB10586, MeT-D1 and MeT-D3 have 1011/1018 identity, while MeT-D1 and the consensus *P. psychrotolerans* methyltransferase sequence have 727/1018 identity, with two short gaps). Domains in the mmpD modules are typically not highly similar – for example, protein alignments of equivalent domains in modules MmpD1 and MmpD3 give identities of: KS 45.6%; DH 28.4%; KR 38.3%; MeT 99.5%; ACP 50.0%.

This finding was curious enough for us to investigate the sequence of other recently published mupirocin cluster sequences. Alignments showed that *P. fluorescens* 2P24 (in accession CP025542) also has a highly-similar repeat between MeT-D1 and MeT-D3, relatively distantly related to the others. The MeT domains of *Pseudomonas* strain QS1027 (in accession PHSU01000014) were less similar to each other (88% identity). However, this is still rather greater than the similarity of either to those of the other strains, implying that they are more closely related to each other than the common ancestor. In general, the similarity of two MeT domains from different clusters is similar to that of the mupirocin clusters as a whole (Table B in S1 Appendix.), confirming that there is no conservation of the repeated sequence, only the presence of repetition.

Table B. Similarity of module 1 and 3 *mmpD* methyltransferase domains, and entire clusters from a variety of strains.

		MeT-D1 [a]<br>Cluster [b]				
**Strain**		**NCIMB 10586**	**NS383 [c]**	**2P24**	**8B**	**QS1027**
**NCIMB10586**<br>**(*P. synxantha*)**	MeT-D3<br>cluster	99.3%<br>(100%)	70.8%<br>68.6%	86.9%<br>84.9%	87.8%<br>85.1%	72.9%<br>70.2%
**NS383**<br>**(*P. psychrotolerans*)**	MeT-Dc [c]<br>cluster	70.8%<br>68.6%	[>99%] [c]<br>(100%)	72.2%<br>68.4%	72.4%<br>68.8%	69.2%<br>69.1%
**2P24**<br>**(*P. fluorescens*)**	MeT-D3<br>cluster	86.7%<br>84.9%	72.1%<br>68.4%	99.7%<br>(100%)	94.6%<br>95.4%	74.4%<br>70.2%
**8B**<br>**(*P. orientalis*)**	MeT-D3<br>cluster	87.4%<br>85.1%	72.1%<br>68.8%	94.0%<br>95.4%	98.0%<br>(100%)	74.0%<br>70.5%
**QS1027**<br>**(*P. sp.)***	MeT-D3<br>cluster	72.4%<br>70.2%	70.1%<br>69.1%	74.0%<br>70.2%	75.1%<br>70.5%	86.2%<br>(100%)

In each cell the domain alignment identity is shown above the cluster alignment identity; both are DNA-level alignments.

[a] Domain alignments used Needle, with the gap-open parameter set to 100 (which has the effect of suppressing gap formation to codon boundaries in all these alignments).

[b] Cluster alignments used Stretcher, with a range from the start codon of *mupZ* to the start codon of *mupI*.

[c] The NS383 domain sequences are derived from a single 'consensus' contig.

The conservation of a repeat in *mmpD* is surprising, in particular because the repeated sequence itself is not conserved between the clusters. This repeat broadly consists of the methyltransferase domains, responsible for bringing in methyl groups onto mupirocin carbon backbone atoms 12 and 8. A 'negative' explanation for this would be that gene conversion of all other functionally related domains in the cluster is deleterious for function, yet not for these methyltransferases. However, we know that recombination between 500 bp identical sequences occurs, at low frequency, in *Pseudomonas* – the widely used "suicide mutagenesis" technique relies on recombination between such repeats to integrate and excise plasmids, and reliably produces recombinants in a plated population of fewer than 10^6 cells [3]. Even though this is a rare event, it would be expected to exert

some selective pressure against such recombinogenic sequences over evolutionary time. It is also hard to explain why a repeat appears to be routinely present in the methyltransferase domains, but not, for example, in the tandem ACPs in module 3 of *mmpA*.

We suggest, therefore, that the existence of a repeat over these regions is somehow favoured. The DNA-level identity of the repeated sequence could be maintained while the sequence diverges from related strains by occasional gene conversion events. We have entertained a number of hypotheses for the creation or maintenance of these repeats:

1) More rapid codon-usage-bias alignment. The mupirocin cluster is occasionally moved to a new strain. After this occurs, there is selection for mutations which improve codon-alignment, and hence expression (both in translation rate and protein folding), in the new host. Regions encoding protein domains where all interactions are equivalent may exchange sequence, increasing the rate of this alignment. This would imply that these regions would have a directional variation in codon usage. Two mupirocin clusters - those found in *P. fluorescens* 2P24 and *P. orientalis* 8B - are closely related and have the full genome sequence available as a single contig. We made a four-way alignment of the 1018 'core' anomalous repeat DNA, further aligned with the corresponding 338 residue peptide sequences. In single-base columns, there are 46 differences where cluster-pairs match (plus 21 single-sequence differences and one mutually different pair). Of these pair-wise differences, 26 are in synonymous codons without other complications.

For each genome, we created a codon usage table. We could then examine how favoured the synonymous codons are for the cognate and opposing strain. Overall, the varied residues are only very slightly more common in the cognate codon usage table for each domain. There is no clear migratory trend – for example, there are two arginine codons, each repeat-set carrying one CGC and one CGT codon. We therefore consider this hypothesis disproven.

2) Gene conversion from related sequence(s) elsewhere in the genome. We considered it possible that the methyltransferase domain may interoperate with well-expressed proteins encoded elsewhere in the genome, and hence increase substrate throughput with gene conversion, after introduction to a new host. However, no similar domains were found in the NCIMB10586 genome by BLAST search, which essentially precludes this.

3) A further type of explanation is that there is a benefit to precisely matching the enzymatic reaction rates, or perhaps the fold, of the module 1 and 3 methyltransferase domains. The implication is that the exact amino-acid sequence encoded is less important than the fact that it matches. Individually, changes away from agreement would not confer enough selective pressure to remove them, however the more distant the domains became, the more advantageous a gene conversion event might be. We do not have any strong evidence for this class of explanation, and consider them unlikely. It may be relevant that the related thiomarinol cluster [4] does not have a repeat over the methyltransferase range. One difference in the product molecule structure is that thiomarinol does not have an epoxide ring, which in mupirocin involves the backbone introduced in module 2. This may be a coincidence, however, because in the current model of mupirocin synthesis, this group is thought to be created at a later stage in the pathway, i.e. after the substrate has passed through module 3 [5].

Whatever the explanation, the gain presumably exceeds the cost of occasional deletion of the sequence between the repeats.

References

1. Zhang Y, Zhang B, Wu H, Wu X, Yan Q, Zhang L-Q. Pleiotropic effects of RsmA and RsmE proteins in Pseudomonas fluorescens2P24. BMC Microbiol 2020;20, 191
2. Midha S, Bansal K, Sharma S, Kumar N, Patil PP, Chaudhry V, Patil PB. Genomic resource of rice seed associated bacteria. Frontiers in Microbiology 2016;6, 1551.
3. Cooper SM, Laosripaiboon W, Rahman AS, Hothersall J, El-Sayed AK, Winfield C, Crosby J, Cox RJ, Simpson TJ, Thomas CM. Shift to Pseudomonic Acid B Production in P. fluorescens NCIMB10586 by Mutation of Mupirocin Tailoring Genes mupO, mupU, mupV, and macpE Chem. Biol. 2005;12, 825–833
4. Fukuda D, Haines AS, Song Z, Murphy A, Hothersall J, Stephens ER, Cox R, Crosby J, Willis C, Simpson TJ, Thomas CM. A natural plasmid uniquely encodes two antibiotic pathways creating a potent hybrid. PLoS ONE 2011;6:e0018031.
5. Gao S, Hothersall J, Wu J, Murphy AC, Song Z, Stephens ER, Thomas CM, Cox RJ, Simpson TJ, Willis CL. The biosynthesis of mupirocin by *Pseudomonas fluorescens* NCIMB 10586 involves parallel pathways. J American Chem Soc 2014; 136:5501–5507.
